# The cGAS–STING Pathway Drives Astrocyte‐Mediated Demyelination in Multiple Sclerosis Through Clusterin Secretion

**DOI:** 10.1002/cns.71091

**Published:** 2026-08-14

**Authors:** Shengfei Hu, Xia Xiao, Xi Cheng, Yiying Huang, Tingting Cui, Shishi Shen, Chunping Cui, Li Xiao, Hanzhang Tan, Wei Qiu, Yipeng Zhao, Rui Li

**Affiliations:** ^1^ Department of Neurology Third Affiliated Hospital of Sun Yat‐Sen University Guangzhou China; ^2^ The Center of Mental and Neurological Disorders Study The Third Affiliated Hospital of Sun Yat‐Sen University Guangzhou China

**Keywords:** astrocyte, cGAS–STING, Clusterin, demyelination, multiple sclerosis

## Abstract

**Background:**

Multiple sclerosis (MS) is a chronic neuroinflammatory disorder characterized by oligodendrocyte injury and demyelination. The disease progresses from peripheral immune attacks to compartmentalized central nervous system (CNS) inflammation, culminating in irreversible neurodegeneration. Although current immunotherapies suppress peripheral relapses, they inadequately address compartmentalized CNS inflammation and progressive neurodegeneration.

**Methods:**

We reanalyzed published single‐nucleus RNA‐seq datasets from human MS lesions. Primary astrocytes, oligodendrocytes, and organotypic cultures were used for in vitro studies. Outcomes were assessed by immunofluorescence, Western blot, qRT‐PCR, RNA‐seq, cell viability assay, and behavioral scoring. The STING inhibitor H‐151 was administered in preventive and therapeutic paradigms.

**Results:**

Single‐nucleus RNA‐seq showed inflammatory astrocytes accumulate preferentially at chronic active lesion edges in MS. These astrocytes exhibited STING pathway activation, coinciding with elevated DNA concentrations in cerebrospinal fluid. Mechanistically, DNA synergized with pro‐inflammatory cytokines to trigger astrocytic STING signaling, driving Clusterin (CLU) secretion that directly induced oligodendrocyte apoptosis and demyelination. Pharmacological inhibition of STING with H‐151 prevented and ameliorated established clinical deficits in experimental autoimmune encephalomyelitis mice.

**Conclusion:**

DNA elevation in inflammatory microenvironments activates the astrocytic STING–CLU axis to promote disease pathogenesis, validating STING targeting as a treatment strategy for MS.

## Graphical abstract

In multiple sclerosis, astrocytes detect extracellular DNA via the cGAS–STING pathway, triggering Clusterin secretion that induces oligodendrocyte apoptosis and demyelination. Pharmacological STING inhibition with H‐151 suppresses this neurotoxic cascade, preserves myelin integrity, and ameliorates clinical disability in experimental autoimmune encephalomyelitis. These findings highlight the astrocytic cGAS–STING–Clusterin axis as a promising therapeutic target for progressive MS.

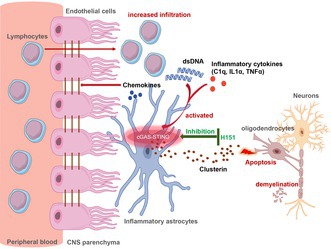

**Abbreviations**
c‐Cas‐3: cleaved Caspase‐3
cGAS–STING: cyclic GMP‐AMP synthase—stimulator of interferon genes
CLU: clusterin
CNS: central nervous system
CSF: cerebrospinal fluid
dsDNA: double‐stranded DNA
EAE: experimental autoimmune encephalomyelitis
ELISA: enzyme‐linked immunosorbent assay
GPX4: glutathione peroxidase 4
HC: healthy controls
MBP: myelin basic protein
MS: multiple sclerosis
NFH: neurofilament high chain
Non‐MS: non‐demyelinating inflammatory disease patients
NPCs: neural progenitor cells
OPC: oligodendrocyte precursor cell
SD: standard deviation

## 1 Introduction

Multiple sclerosis (MS) is an autoimmune disorder characterized by neuroinflammation and demyelination within the central nervous system (CNS) [1]. Current disease‐modifying therapies have achieved remarkable success in limiting relapses by targeting peripheral immune cell infiltration [2]. However, a critical unmet clinical need persists: progressive disability continues to accumulate in many patients despite suppression of peripheral inflammation, driven by relentless CNS‐intrinsic inflammation, cumulative demyelination, and neurodegeneration [3]. This clinical reality exposes a fundamental gap: Therapeutic strategies targeting the intrinsic inflammatory machinery of the CNS parenchyma remain virtually nonexistent, underscoring an urgent need to decipher the molecular mechanisms governing CNS‐intrinsic neuroinflammation [4].

Within the CNS parenchyma, astrocytes have emerged as master regulators of tissue homeostasis and innate immune responses [5]. Under pathological conditions, astrocytes undergo reactive transformations involving morphological alterations, molecular phenotypic shifts, and functional remodeling [6, 7]. This dynamic process is implicated in MS pathogenesis, although the underlying mechanisms remain incompletely defined. Recent studies indicate that astrocytes can exacerbate oligodendrocyte apoptosis and myelin injury by secreting pro‐inflammatory cytokines and chemokines in neuroinflammatory microenvironments [8, 9]. Yet, the fundamental question of how astrocytes detect danger signals within the inflamed CNS microenvironment and through what molecular logic they convert these sensing events into neurotoxic outputs remains entirely unresolved.

During MS progression, inflammation‐associated cellular damage can be accompanied by DNA release. The DNA‐sensing cGAS–STING pathway offers an attractive entry point for understanding astrocyte‐mediated danger signal detection. As the principal sensor for double‐stranded DNA (dsDNA), this pathway initiates innate immune responses through production of type I interferons and pro‐inflammatory cytokines [10, 11, 12]. Notably, this pathway exhibits dual roles in neuroinflammation. In MS, recent research demonstrated that cGAMP‐encapsulated microparticles activate STING signaling, enhance ERK phosphorylation, and suppress autoreactive T cells, thereby conferring therapeutic benefits [13]. Conversely, in progressive and relapsing–remitting MS patients, neuronal depletion of endoplasmic reticulum Ca^2+^ releases STIM1‐mediated inhibition of STING, triggering autophagic degradation of glutathione peroxidase 4 (GPX4), culminating in ferroptosis and accelerated disease progression [14]. As the predominant glial population, astrocytes have also been implicated in the pathology of Parkinson's disease, amyotrophic lateral sclerosis, and Alzheimer's models through mechanisms mediated by cGAS–STING activation [15, 16, 17]. However, whether astrocytes engage this pathway to sense CNS‐derived DNA danger signals, and what specific downstream mechanisms link such sensing to myelin injury, are unknown.

Here, we demonstrate that astrocytes function as pivotal DNA sensors within the inflamed lesion and drive a robust neurotoxic transformation. Through integrated transcriptomic, snRNA‐seq, and functional analyses, we identify Clusterin (CLU) as a distinct, non‐canonical effector of astrocytic cGAS–STING signaling that directly mediates oligodendrocyte apoptosis and demyelination. Pharmacological targeting of STING with the brain‐penetrant inhibitor H‐151 abolishes astrocytic CLU secretion, attenuates CNS inflammation, and preserves myelin integrity in both preventive and therapeutic treatment paradigms. Collectively, these findings demonstrate astrocytic DNA sensing through the cGAS–STING–CLU axis as a central mechanism linking innate immune surveillance to CNS‐intrinsic neurodegeneration which may provide a theoretical foundation for developing precision therapies targeting glial immunomodulation in MS.

## 2 Materials and Methods

### 2.1 Single‐Cell Data Analysis

The published single‐cell dataset (GSE180759 [18], GSE118257 [19], GSE179590 [20]) was reanalyzed using Seurat v.5.0.0 in R v.4.3.1 [21]. The filtering criteria were as follows: (1) Unique molecular identifier (UMI) count > 1000; (2) 200 < genes detected per cell < 7000; (3) Mitochondrial gene percentage < 20%; and (4) Red blood cell gene expression < 5% of total genes.

For dimensionality reduction and clustering analysis of snRNA‐seq data, we first performed log‐normalization to account for technical variability across samples. Highly variable genes (HVGs) were identified and selected to capture major biological signals, while mitochondrial gene expression proportions were regressed out during normalization to mitigate potential confounding effects from cellular stress or death.

Principal component analysis (PCA) was applied for initial linear dimensionality reduction to capture primary sources of transcriptional variation. To integrate multi‐sample datasets and correct batch effects, we employed the Harmony algorithm, enabling robust downstream dimensionality reduction and unsupervised clustering. Uniform manifold approximation and projection (UMAP) was subsequently used for non‐linear dimensionality reduction to visualize high‐dimensional transcriptomic relationships in two‐dimensional space. Cell population distributions and phylogenetic relationships were visualized using UMAP embeddings and hierarchical clustering dendrograms.

Cluster‐specific gene expression profiles were systematically examined. Clusters exhibiting similar transcriptional signatures were merged to optimize resolution. Cell type identities were manually annotated based on established lineage‐specific marker genes and canonical transcriptional signatures, with classifications validated through differential expression analysis and reference dataset comparison in subsequent steps.

### 2.2 Calculation of Gene Signature Scores

To quantify cGAS‐STING pathway activation and lymphocyte infiltration at the single‐cell level, we computed module scores using the Seurat AddModuleScore function. For the cGAS‐STING signature, we curated a comprehensive gene set comprising core signaling molecules (CGAS, STING1/TMEM173, TBK1, IRF3, IKBKE, NFKB1) and downstream interferon‐stimulated genes (STAT1, STAT2, IRF7, ISG15, CXCL10, IFIT1/2/3, OASL2, IFI44, IFIH1, DDX58, RTP4, ICAM1, CCL2). For the lymphocyte infiltration signature, we selected lineage‐defining markers including CD3D, CD3E, TRAC, TRBC2, IL7R, CD4, and CD8A. Module scores were calculated as the average expression of each gene set per cell, with expression values centered and scaled across the dataset. Higher scores indicate enhanced pathway activation or immune infiltration, respectively.

### 2.3 Experimental Animals

All animal experiments were performed in conformity to the guidelines of the National Institutes of Health on the care and use of animals. Specific pathogen‐free female C57BL/6 wild‐type mice (6–8 weeks old) were purchased from Beijing Vital River Laboratory Animal Technology Co. Ltd. All animals were housed at the Animal Experiment Center of South China Agricultural University under controlled temperature and humidity with a 12‐h light/dark cycle and provided ad libitum access to food and water.

### 2.4 Establishment of the EAE Model

Female C57BL/6 wild‐type mice (7 weeks old) were immunized subcutaneously with 200 μg myelin oligodendrocyte glycoprotein peptide 35–55 (MOG_₃₅₋₅₅_) emulsified in complete Freund's adjuvant (1:1 volume ratio). Pertussis toxin (150 ng per mouse) was administered intraperitoneally on days 0 and 2 post‐immunization.

After immunization, mice were observed daily using a double‐blind method, and behavioral scores were recorded according to the following criteria: (1) tail paralysis; (2) mild hind limb paralysis; (3) complete hind limb paralysis; (4) complete hind limb paralysis with forelimb paralysis; (5) terminal stage.

For the study of the STING inhibitor H151, mice were divided into four groups according to treatment: the WT group, the EAE control group, the preventive treatment group (H151 750 nmol/mouse daily intraperitoneal injection from day 8 post‐immunization until disease onset), and the therapeutic treatment group (H151 750 nmol/mouse daily intraperitoneal injection from the onset of clinical symptoms until the clinical progression of the EAE control group was halted).

### 2.5 Clinical Samples

Cohort recruitment spanned January 2023 to September 2024 and comprised three groups: patients with multiple sclerosis (MS), patients with non‐demyelinating inflammatory neurological diseases (Non‐MS), and healthy controls (HC).

### 2.6 Primary Cell Culture

Primary Astrocyte Culture: Primary cortical astrocytes were isolated from postnatal day 0 (P0) C57BL/6J mouse pups. Following meninges removal, cortical tissue was digested with 0.125% trypsin–EDTA (37°C, 15 min). Digestion was quenched with astrocyte growth medium, and the cell suspension was filtered through a 70 μm cell strainer. Cells were plated into poly‐D‐lysine‐coated T75 flasks (two brains per flask) in astrocyte medium [DMEM/F12 supplemented with 10% fetal bovine serum, 1% non‐essential amino acids, 1.6 mM L‐glutamine, 0.2 mM sodium pyruvate, and 1% penicillin–streptomycin]. Medium was replenished every 48 h. Upon reaching confluence (day 7), microglial and oligodendrocyte precursor contaminants were removed by differential adhesion (shaking at 200 rpm, 37°C, 18 h). Purified astrocytes were used within three passages.

Primary Oligodendrocyte Culture: Oligodendrocyte lineage cells were derived from neural progenitor cells (NPCs) isolated from P0 mouse cortex. Cortical tissue was dissociated with trypsin (room temperature, 15 min), and NPCs were expanded as neurospheres in NPC medium [DMEM/F12, 2% B27, 20 ng/mL EGF, 20 ng/mL bFGF, 1.6 mM L‐glutamine, 1% penicillin–streptomycin], with passaging every 3 days. Third‐passage neurospheres were dissociated and plated onto poly‐D‐lysine‐coated (0.1 mg/mL) 24‐well plates in OPC induction medium [DMEM/F12, 2% B27, 10 ng/mL bFGF, 10 ng/mL PDGF‐AA, 1% penicillin–streptomycin]. After 48 h, medium was switched to differentiation medium [neurobasal medium, 2% B27, 1% penicillin–streptomycin] for 5 days to generate mature oligodendrocytes.

### 2.7 Organotypic Slice Culture

Cerebellar tissue was harvested from P7 C57BL/6 mice, and coronal cerebellar sections (350 μm) were prepared using an McIlwain tissue slicer. Three slices were placed into the upper chamber of a 12‐well culture plate, with 500 μL culture medium added to the lower chamber per well. The culture medium consisted of 50% MEM, 25% HBSS, 25% horse serum, 28 mM D‐glucose, 2 mM glutamine, and 1% penicillin–streptomycin. Slices were maintained at 37°C and 5% CO_2_. Medium was changed the following day and subsequently every 2 days. Experiments were performed on day 7.

### 2.8 Western Blotting

Cells were lysed in RIPA buffer containing protease and phosphatase inhibitors. Spinal cord tissue was homogenized in lysis buffer and sonicated. Lysates were incubated on ice (30 min) and clarified by centrifugation (17,000 × g, 5 min, 4°C). Protein concentration was determined by BCA assay. Samples were denatured (95°C, 15 min), resolved by SDS‐PAGE (10–30 μg per lane, 110 V), and transferred to PVDF membranes (200 mA, 2 h). Membranes were blocked with 5% non‐fat milk in TBST (1 h, room temperature), incubated with primary antibodies (overnight, 4°C), washed, and probed with HRP‐conjugated secondary antibodies (1 h, room temperature). Signals were detected by enhanced chemiluminescence and captured using a ChemiDoc MP imaging system (Bio‐Rad).

### 2.9 Immunofluorescence Staining

Mice were transcardially perfused with PBS followed by 4% paraformaldehyde. Spinal cords were harvested, cryoprotected, and sectioned coronally (20 μm). Tissue sections, cultured cells, and organotypic slices were fixed, permeabilized (0.25% Triton X‐100, 20 min), and blocked (3% BSA, 1 h). Primary antibody incubation was performed overnight at 4°C (tissue/cells) or 48 h at 4°C (organotypic slices). Following PBS washes, samples were incubated with fluorophore‐conjugated secondary antibodies (1 h, room temperature), mounted with DAPI‐containing antifade medium, and imaged by fluorescence microscopy.

### 2.10 Cell Viability Assay

Cell viability was assessed using the Cell Counting Kit‐8 (CCK‐8) according to the manufacturer's protocol. CCK‐8 reagent (10 μL per 100 μL medium) was added to each well of a 96‐well plate, incubated (1–4 h, 37°C), and absorbance at 450 nm was measured using a microplate reader (BioTek).

### 2.11 Enzyme‐Linked Immunosorbent Assay (ELISA)

Serum and cerebrospinal fluid (CSF) samples were collected from MS patients, Non‐MS patients, and healthy controls. Serum was isolated by centrifugation (3000 × g, 15 min, 4°C) and stored at −80°C until analysis. Concentrations of human C3, GFAP, CCL2, CCL5, dsDNA, and CLU were quantified using commercial ELISA kits (Meimian Biotechnology) following the manufacturer's instructions.

### 2.12 Quantitative Real‐Time Polymerase Chain Reaction (RT‐PCR)

Total RNA was extracted using TRIZOL reagent according to the manufacturer's protocol. RNA concentration was measured using a NanoDrop spectrophotometer. cDNA was synthesized using a fast reverse transcription kit following the manufacturer's instructions, and RT‐qPCR was performed on a QuantStudio 5 (ABI) quantitative PCR instrument. Data were normalized as the difference in CT values relative to GAPDH (Delta CT log_2_) and compared with the control group mean. The specific primers (synthesized by GENEWIZ, Suzhou, China) were used as follows:

**Table:** 

Gene	Forward primer	Reverse primer
*Ccl2*	CCTGCTGCTACTCATTCACCA	ATTCCTTCTTGGGGTCAGCA
*Ccl5*	GTGCCCACGTCAAGGAGTAT	CTCTGGGTTGGCACACACTT
*Clu*	GATGATCCACCAGGCTCAACAG	ACACAGTGCGGTCATCTTCACC
*Gapdh*	GAAGGTCGGTGTGAACGGAT	AATCTCCACTTTGCCACTGC

### 2.13 Bulk RNA Sequencing (RNA‐Seq)

Total RNA from cultured primary astrocytes was extracted using TRIzol reagent (Invitrogen) according to the manufacturer's instructions. RNA quality and quantity were assessed using a NanoDrop spectrophotometer and an Agilent 2100 Bioanalyzer (Thermo Fisher Scientific, USA). Strand‐specific cDNA libraries were constructed via phi29 polymerase amplification and DNA nanoball (DNB) preparation, generating more than 300 copies per molecule. DNBs were loaded onto a patterned nanoarray, and 50‐bp single‐end sequencing was performed on the BGIseq500 platform.

Raw reads were quality‐filtered using SOAPnuke, including removal of reads containing adapter sequences, low‐quality bases (quality score ≤ 5), and reads with > 5% unknown bases (N). Clean reads were saved in FASTQ format and aligned to the reference genome (GCF_000001635.27_GRCm39) using HISAT2 and Bowtie2. Gene expression levels were quantified using RSEM.

RNA‐seq data were organized as a data frame with genes as rows and samples as columns. Raw data were aligned, quality‐controlled, and normalized to generate a raw count expression matrix. To account for sequencing‐depth differences, FPKM values were corrected for gene length. Log‐transformed FPKM values were used to reduce bias and satisfy assumptions for downstream analyses. A design matrix was constructed using factor variables. The voom function was applied for variance‐stabilizing transformation, weighted standard error estimation, and mean–variance trend plotting. Linear modeling (lmFit) based on the design matrix was used to evaluate gene expression changes across conditions, and Bayesian statistics (eBayes) were applied to improve statistical power and calculate significance between groups. Differentially expressed genes (DEGs) were selected using the Benjamini–Hochberg (BH) method for multiple‐testing correction and ranked by *p*‐value.

Genes with significant changes between groups were defined using a statistical significance threshold (*p*‐value) and a fold‐change threshold (log_2_ fold change, logFC), and classified as upregulated, downregulated, or stable. Volcano plots were generated using ggplot2 with log_2_ fold change on the x‐axis and −log_10_(*p*‐value) on the y‐axis.

For Gene Ontology (GO) enrichment analysis, significantly upregulated and downregulated genes were identified from the differential expression results. Because GO analysis typically uses Entrez IDs, the bitr function was used to convert gene names from SYMBOL to ENTREZ IDs. Mouse annotations were obtained using the org.Mm.eg.db database. GO enrichment analysis was performed for biological processes (BP) using enrichGO with parameters *p*valueCutoff and *q*valueCutoff. Relevant pathways were selected, and significant pathways were extracted for visualization.

### 2.14 Statistical Analysis

All statistical analyses were performed using GraphPad Prism (version 9.0). Data are presented as mean ± standard deviation (SD) from at least three independent experiments. Normality was assessed using the Shapiro–Wilk test. For comparisons between two groups, an unpaired two‐tailed *t*‐test was used when data were normally distributed (error bars represent SD); otherwise, non‐parametric tests were applied. For comparisons among three or more groups, one‐way ANOVA was used when data met normality assumptions (without pairing or matching), followed by multiple‐comparison testing (Dunnett or Bonferroni), with error bars representing SD. If normality assumptions were not met, non‐parametric tests were used. EAE clinical scores were compared using the Mann–Whitney *U* test, with error bars representing the standard error of the mean (SEM). A 95% confidence interval was applied, and *p* < 0.05 was considered statistically significant (*p < 0.05, **p < 0.01, ***p < 0.001, ****p < 0.0001; ns, not significant).

## 3 Results

### 3.1 Inflammatory Astrocytes Accumulate at Chronic Active Lesion Edges in Multiple Sclerosis

To investigate the molecular mechanisms driving astrocyte‐mediated neuroinflammation in MS, we analyzed snRNA‐seq datasets from human MS white matter lesions (GSE180759, GSE118257, GSE179590). Unsupervised clustering identified major CNS cell populations, including homeostatic astrocytes (Ast), reactive astrocytes (ReactiveAst), oligodendrocytes (Oligo), neurons, oligodendrocyte precursor cells (OPC), lymphocytes (Lym), endothelial cells (Endo), pericytes (Peri), and ependymal cells (Epen) (Figure 1A,B). Notably, reactive astrocytes exhibited upregulation of complement component C3 and glial fibrillary acidic protein (GFAP) [22, 23], consistent with an inflammatory phenotype (Figure 1C). Cell composition analysis revealed a marked expansion of reactive astrocytes within chronic active lesion edges (SPMS_CA_LE) and active lesion (SPMS_A) compared to periplaque white matter and healthy controls (Figure 1D,E). Violin plots further revealed elevated expression of both GFAP and C3 specifically within astrocytes derived from SPMS_CA_LE compared to control white matter (Figure 1F,G). To validate these transcriptomic observations at the protein level, we measured astrocyte activation markers in cerebrospinal fluid (CSF) from an independent cohort of MS patients. ELISA confirmed significantly elevated GFAP and complement C3 protein levels in MS patients compared to non‐inflammatory neurological controls (Non‐MS) (Figure 1H,I). Moreover, serum C3 concentrations correlated positively with expanded disability status scale (EDSS) scores (*R* = 0.6144, *p* < 0.0001), indicating that astrocytic activation markers reflect clinical disease severity (Figure 1J). These data establish that inflammatory astrocytes, characterized by C3 and GFAP upregulation, accumulate preferentially at active demyelinating lesion edges and that their activation signatures are detectable in biofluids, correlating with neurological disability.

**FIGURE 1:**
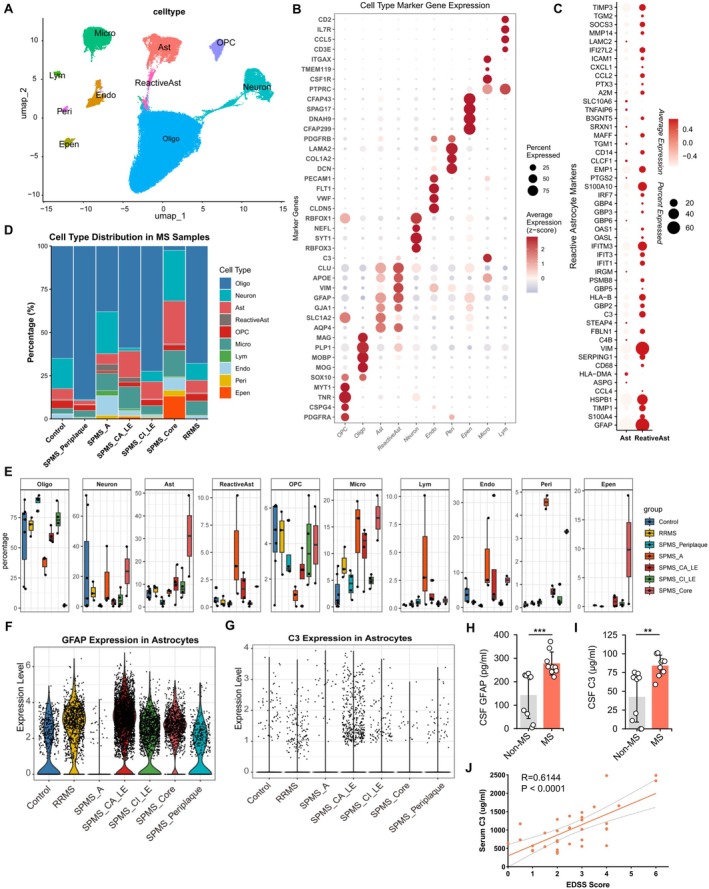
Accumulation of inflammatory astrocytes at chronic active lesion edges correlates with clinical severity in multiple sclerosis. (A) Uniform manifold approximation and projection (UMAP) visualization of cell types identified from human MS white matter snRNA‐seq dataset (GSE180759, GSE118257, GSE179590), including microglia (Micro), astrocytes (Ast), reactive astrocytes (ReativeAst), oligodendrocytes (Oligo), neurons, oligodendrocyte precursor cells (OPC), lymphocytes (Lym), endothelial cells (Endo), pericytes (Peri), and ependymal cells (Epen). snRNA‐seq data includes normal‐like white matter (SPMS_Periplaque, *n* = 18,814 nuclei), inactive lesion edges (SPMS_CI_LE, *n* = 14,372 nuclei), active LE (SPMS_CA_LE, *n* = 19,961 nuclei), and lesion cores (SPMS_Core, *n* = 4161 nuclei) from individuals with SPMS, as well as white matter from individuals with RRMS (*n* = 13,297 nuclei) and healthy control (*n* = 10,082 nuclei). (B) Dot plot showing expression of canonical marker genes across identified cell types. Dot size represents percentage of expressing cells; color intensity indicates average expression level. (C) Expression of reactive astrocyte markers in astrocyte subpopulations. (D) Stacked bar chart depicting cell type composition across different lesion locations. (E) Box plots showing relative proportions of major cell types across different lesion locations. (F‐G) Violin plots showing single‐cell expression analysis of GFAP (F) and C3 (G) within astrocyte subpopulations across different lesion locations. (H‐I) Quantification of GFAP (H) and C3 (I) protein levels in cerebrospinal fluid (CSF) from MS patients (*n* = 9) versus non‐inflammatory neurological disease controls (Non‐MS; *n* = 9) by ELISA. (J) Correlation analysis between serum C3 concentrations and Expanded Disability Status Scale (EDSS) scores in MS patients (*n* = 34), showing a significant positive association (Spearman correlation).

### 3.2 Inflammatory Astrocytes Exhibit Neurotoxic Potential via cGAS‐STING Signaling

To validate these clinical findings in an animal model, we examined spinal cord white matter from EAE mice and found increased expression of GFAP and C3 compared to controls (Figure 2A,B). To model inflammatory astrocyte activation in vitro, we stimulated primary mouse astrocytes with pro‐inflammatory factors (IL‐1α, TNF‐α, and C1q) previously established to induce the neuroinflammatory phenotype [22]. This stimulation paradigm significantly upregulated GFAP and C3 expression (Figure 2C,D). To identify regulatory pathways associated with inflammatory astrocytes in MS, we performed RNA sequencing of primary astrocytes following pro‐inflammatory stimulation. GO enrichment analysis of upregulated genes showed significant enrichment of innate immune response, viral response, DNA damage response, and type I interferon pathways (Figure 2E and Figure S1A). Western blotting and immunofluorescence confirmed cytokine‐induced upregulation of cGAS and phosphorylated STING (P‐STING), indicative of pathway activation (Figure 2F,G and Figure S1B,C).

**FIGURE 2:**
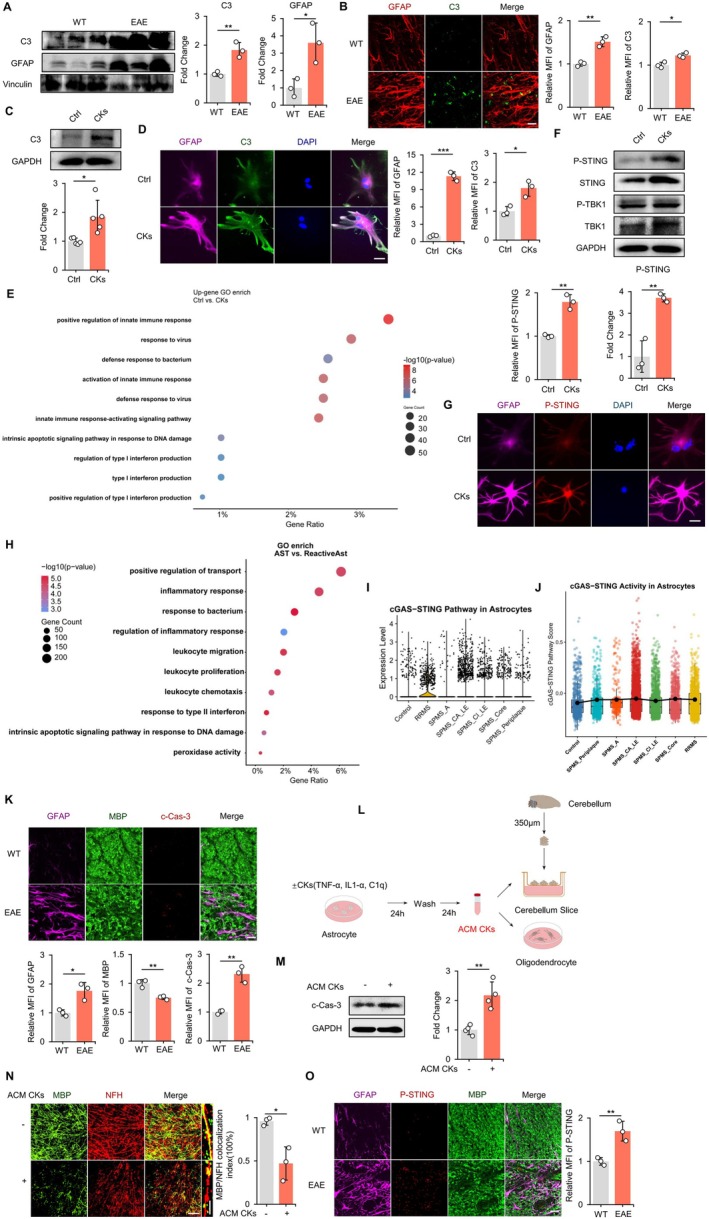
Activation of the cGAS–STING pathway in inflammatory astrocytes within multiple sclerosis lesions (A) Representative Western blot images and densitometric quantification of C3 and GFAP protein expression in spinal cord lysates from WT and EAE mice. (B) Representative confocal immunofluorescence images of GFAP (red) and C3 (green) staining in lumbar spinal cord white matter from WT and EAE mice (scale bars, 20 μm), with quantification of relative mean fluorescence intensity (MFI). (C–D) Primary mouse astrocytes were treated for 24 h with PBS (Ctrl) or pro‐inflammatory cytokines (CKs). (C) Western blot analysis of C3 protein expression with GAPDH as loading control, and densitometric quantification (*n* = 3 biological replicates). (D) Representative confocal images showing immunofluorescence staining for GFAP (magenta) and C3 (green) (scale bars, 20 μm) with quantification of relative MFI. (E) Gene Ontology (GO) enrichment analysis of upregulated genes in CKs‐stimulated versus control astrocytes. Bubble size represents gene count; color intensity indicates‐log10(*p*‐value). (F) Western blot analysis of cGAS–STING pathway activation in primary astrocytes following 24‐h CKs stimulation. Right panel shows quantification of P‐STING levels (*n* = 3). (G) Representative confocal immunofluorescence images of GFAP (magenta) and P‐STING (red) in primary astrocytes with or without CKs stimulation (scale bars, 20 μm), with quantification of P‐STING MFI. (H) Gene Ontology (GO) enrichment analysis of biological processes upregulated in reactive astrocytes compared to homeostatic astrocytes. (I) Violin plots showing expression levels of cGAS–STING pathway components (CGAS, STING1/TMEM173, TBK1, IRF3, IKBKE, NFKB1) in astrocytes versus other CNS cell types. (J) cGAS–STING pathway activity scores in astrocytes across different lesion locations. (K) Representative confocal images of GFAP, MBP, and cleaved Caspase‐3 (c‐Cas‐3) staining in the lumbar white matter of WT and EAE mice (scale bar, 20 μm), with quantitative analysis of relative fluorescence intensity. (L) Schematic of conditioned‐medium protocol: Primary astrocytes were stimulated with PBS or pro‐inflammatory factors for 24 h, washed, cultured for another 24 h, and the supernatant was applied to primary oligodendrocytes and cerebellar slices for 24 h. (M) Western blot of c‐Cas‐3 in primary oligodendrocytes after 24 h treatment with astrocyte‐conditioned medium, with densitometric quantification. (N) Confocal images of NFH and MBP co‐localization in cerebellar organotypic slices treated for 24 h with astrocyte‐conditioned medium (left scale bar, 50 μm; right, 20 μm), with quantitative analysis of the NFH/MBP co‐localization index. (O) Representative images and quantification of P‐STING in GFAP^+^ astrocytes within demyelinated regions of EAE mouse spinal cord (scale bar, 20 μm). *p < 0.05, **p < 0.01, ***p < 0.001, ****p < 0.0001.

Given the enrichment of DNA damage and interferon responses in stimulated astrocytes, we next examined whether the cGAS‐STING cytosolic DNA‐sensing pathway is activated in reactive astrocytes within human MS lesions. Analysis of our snRNA‐seq datasets revealed that differentially expressed genes in reactive astrocytes were significantly enriched for biological processes including intrinsic apoptotic signaling in response to DNA damage, response to bacterium, and inflammatory response (Figure 2H). We then examined the expression dynamics of individual cGAS‐STING pathway components across lesion locations. Violin plots showed elevated expression specifically within SPMS_CA_LE compared to control white matter (Figure 2I), with periplaque regions exhibiting levels comparable to controls, suggesting that pathway activation is spatially restricted to active demyelinating areas rather than diffuse. To quantify pathway activity at the single‐cell level, we computed integrated cGAS‐STING module scores. Boxplot visualization with superimposed individual data points demonstrated significantly elevated pathway scores in astrocytes derived from SPMS_CA_LE relative to all other lesion locations (Figure 2J). These findings suggest a spatial association between cGAS‐STING activation and active tissue injury.

To assess functional consequences, we tested whether inflammatory astrocytes exert neurotoxic effects on oligodendrocytes. In EAE spinal cords, cleaved Caspase‐3 (c‐Cas‐3) immunoreactivity localized to GFAP‐positive astrocyte‐adjacent cells within demyelinated regions (Figure 2K). Conditioned medium from cytokine‐stimulated astrocytes induced significant apoptosis in primary oligodendrocytes, evidenced by increased c‐Cas‐3 expression (Figure 2L,M). Furthermore, organotypic cerebellar slice cultures exposed to astrocyte‐conditioned medium exhibited disrupted myelin basic protein (MBP) and neurofilament high chains (NFHs) colocalization, indicating myelin structural damage (Figure 2L,N). Notably, P‐STING and cGAS immunoreactivity colocalized with GFAP‐positive astrocytes within demyelinated regions of EAE spinal cords (Figure 2O and Figure S1D), establishing an association between cGAS–STING activation and oligodendrocyte injury.

### 3.3 DNA Synergizes With Inflammatory Signals to Amplify Astrocytic STING Responses

Given that cGAS‐STING activation typically requires cytosolic DNA sensing, we investigated whether extracellular DNA—released during inflammatory tissue damage—could exacerbate astrocyte activation. ELISA quantification revealed significantly elevated cell‐free double‐stranded DNA (dsDNA) concentrations in both CSF and serum from MS patients compared to controls (Figure 3A). Serum dsDNA levels correlated positively with disability severity (Figure 3B).

**FIGURE 3:**
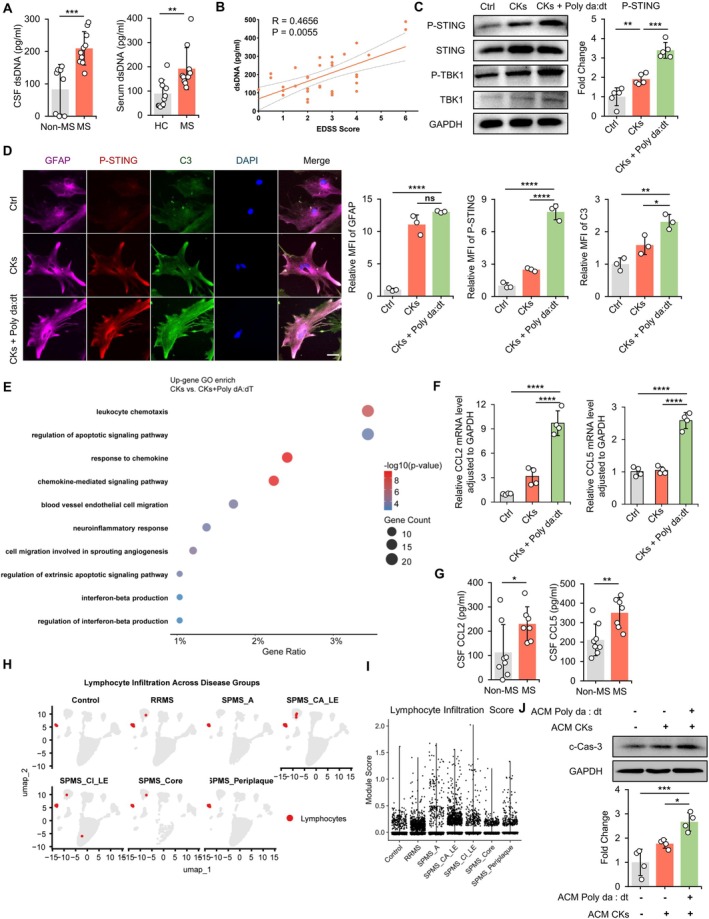
dsDNA synergizes with pro‐inflammatory cytokines to amplify astrocytic STING signaling and neuroinflammatory responses. (A) Quantification of double‐stranded DNA (dsDNA) concentrations in CSF (left) and serum (right) from MS patients versus controls (Non‐MS or healthy controls, HC). (B) Correlation analysis between serum dsDNA levels and EDSS scores. (C) Western blot analysis of cGAS‐STING pathway activation in primary mouse astrocytes stimulated for 24 h with CK followed by 3 h treatment with poly (dA:DT) (800 ng/mL) to simulate DNA exposure. Quantification of P‐STING fold change relative to control is shown (right). (D) Representative confocal immunofluorescence images of primary astrocytes stained for GFAP (magenta), P‐STING (red), and complement component C3 (green), under indicated stimulation conditions (scale bars, 20 μm). Right panels display quantification of relative MFI for each marker. (E) GO enrichment analysis of genes upregulated by combined CKs + poly(dA:DT) stimulation versus CKs alone. (F) Quantitative RT‐PCR analysis of chemokine mRNA expression (Ccl2, Ccl5) in astrocytes under indicated stimulation conditions, normalized to *Gapdh*. (G) ELISA quantification of CCL2 and CCL5 protein concentrations in CSF from MS patients (*n* = 10) versus non‐MS controls (*n* = 10). (H‐I) snRNA‐seq analysis of lymphocyte infiltration across different lesion locations. (H) UMAP visualization showing lymphocyte distribution in control white matter, RRMS, and SPMS subtypes (SPMS_A, SPMS_CA_LE, SPMS_CI_LE, SPMS_Core, SPMS_Periplaque). (I) Violin plots quantifying lymphocyte infiltration scores across different lesion locations. (J) Western blot analysis of c‐Cas‐3 in primary oligodendrocytes treated for 24 h with ACM from the indicated stimulation groups. GAPDH served as loading control. *p < 0.05, **p < 0.01, ***p < 0.001, ****p < 0.0001.

To test this mechanistically, we stimulated primary astrocytes with pro‐inflammatory cytokines followed by poly(dA:dT) to mimic exogenous DNA exposure. Western blotting showed that combined stimulation with poly(dA:dT) and pro‐inflammatory factors significantly enhanced STING pathway activation at 3 h (Figure 3C and Figure S2A). C3 expression was further upregulated in the combined‐stimulation group (Figure 3D and Figure S2B). RNA‐seq analysis of co‐stimulated astrocytes revealed prominent upregulation of neuroinflammatory response pathways, particularly chemokine signaling networks (Figure 3E). Quantitative RT‐PCR confirmed significant induction of Ccl2 and Ccl5 mRNA (Figure 3F), with corresponding elevation of CCL2 and CCL5 protein levels in MS patient CSF (Figure 3G).

UMAP visualization showed the distribution of infiltrating lymphocytes across different lesion locations (Figure 3H), while dot plot demonstrated increased lymphocyte infiltration scores specifically within SPMS_CA_LE (Figure 3I). These snRNA‐seq findings suggest immune cell recruitment as a downstream consequence. Functionally, conditioned medium from astrocytes receiving combined cytokine and DNA stimulation exerted enhanced neurotoxicity, inducing higher levels of oligodendrocyte apoptosis (c‐Cas‐3) and more severe myelin disruption in cerebellar slices than medium from singly stimulated astrocytes (Figure 3J and Figure S2C).

### 3.4 CLU Secretion as a Neurotoxic Effector Downstream of Astrocytic STING Activation

To identify specific mediators responsible for astrocyte‐induced neurotoxicity, we intersected gene sets enriched in three biological processes upregulated by combined cytokine and DNA stimulation: “neuroinflammatory response,” “intrinsic apoptotic signaling pathway in response to DNA damage,” and “intrinsic apoptotic signaling pathway.” This analysis identified Clu (encoding Clusterin) as the sole gene encoding a secreted protein common to all three pathways (Figure 4A). Analysis of the bulk RNA‐seq data confirmed that among the top 20 upregulated genes, *Clu* was the gene previously implicated in oligodendrocyte injury and apoptosis that also encodes a classically secreted protein (Figure S3A). We next profiled canonical STING downstream secreted factors in astrocyte‐conditioned medium by ELISA. Consistent with the canonical STING–IRF3 axis, IFN‐β was the most abundantly secreted product; however, among these secreted factors, CLU exhibited the greatest fold‐change increase (Figure S3B and Figure 4H). To directly compare the functions of IFN‐β and CLU, we assessed the effects of recombinant IFN‐β, recombinant CLU, and their combination on primary oligodendrocyte viability. While IFN‐β induced a modest reduction in oligodendrocyte viability, CLU caused significantly more severe injury. Notably, combined treatment with IFN‐β and CLU exerted additive neurotoxicity (Figure S3C), suggesting cooperative rather than redundant action. These data establish CLU as the dominant neurotoxic executioner within the STING‐driven secretome. snRNA‐seq analysis confirmed elevated CLU expression in reactive astrocytes compared to homeostatic astrocytes, with highest expression observed in chronic active lesion edges (SPMS_CA_LE) (Figure 4B,C). CellChat analysis revealed rewiring of intercellular communication networks in SPMS_CA_LE. Specifically, reactive astrocytes exhibited markedly enhanced outgoing CLU signaling compared to control white matter, indicating increased secretion of this neurotoxic factor. Correspondingly, oligodendrocytes displayed prominent incoming CLU signals in SPMS_CA_LE, establishing a selective communication axis wherein astrocyte‐derived CLU preferentially targets myelinating cells. This altered signaling pattern suggests that CLU mediates directed astrocyte‐to‐oligodendrocyte communication, contributing to myelin damage in actively demyelinating regions (Figure 4D,E).

**FIGURE 4:**
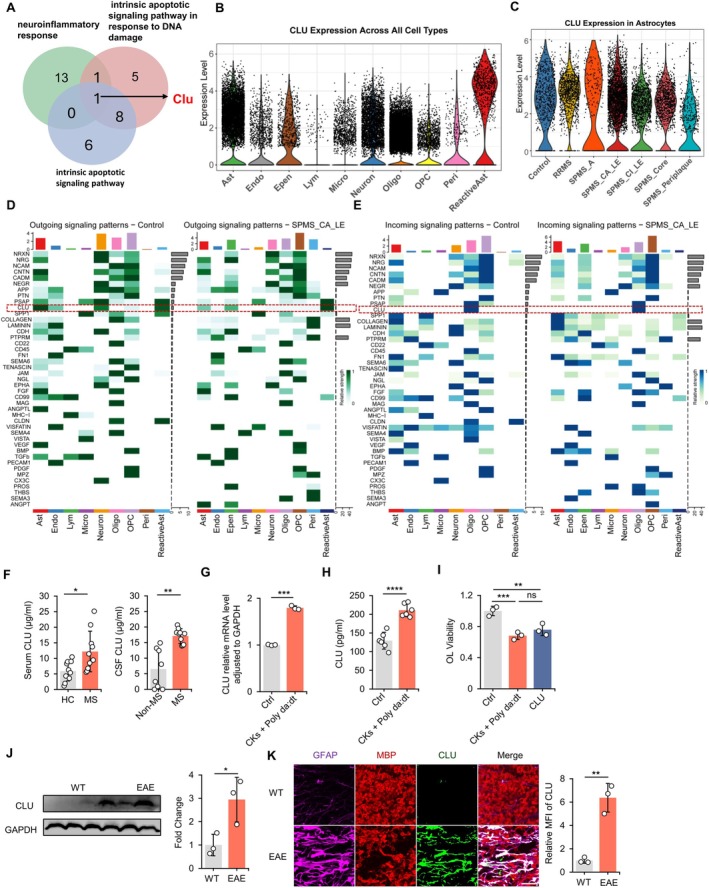
Clusterin (CLU) is identified as a neurotoxic effector downstream of astrocytic cGAS–STING signaling (A) Venn diagram identifying the intersection of genes enriched in three biological processes upregulated in astrocytes co‐stimulated with CKs and poly(dA:DT): “neuroinflammatory response”, “intrinsic apoptotic signaling pathway in response to DNA damage” and “intrinsic apoptotic signaling pathway”. Clu (encoding Clusterin) is the sole gene encoding a secreted protein present in all three pathways. (B–C) Violin plots showing single‐cell expression analysis of CLU across all CNS cell types (B) and specifically within astrocyte subpopulations across different lesion locations (C). (D‐E) CellChat analysis of outgoing (D) and incoming (E) signaling patterns in control versus chronic active lesion edge (SPMS_CA_LE), highlighting altered CLU‐mediated communication (red dashed boxes). Color intensity indicates relative signaling strength. (F) Quantification of CLU protein concentrations in serum (left) and CSF (right) from MS patients versus HC or non‐MS controls, measured by ELISA. (G) Quantitative RT‐PCR analysis of Clu mRNA expression in primary mouse astrocytes stimulated with CKs + poly(dA:DT) versus control, normalized to Gapdh. (H) ELISA measurement of secreted CLU protein in ACM from stimulated versus control astrocytes. (I) Cell viability assessment (CCK‐8 assay) of primary oligodendrocytes treated for 24 h with ACM from stimulated astrocytes or recombinant CLU protein (100 ng/mL) as positive control. (J) Western blot analysis and densitometric quantification of CLU protein expression in spinal cord lysates from WT versus EAE mice. (K) Representative confocal immunofluorescence images of lumbar spinal cord white matter from WT and EAE mice stained for GFAP (magenta), myelin basic protein (MBP, red), and CLU (green), with merged images (scale bars, 20 μm). Right panel shows quantification of CLU MFI. *p < 0.05, **p < 0.01, ***p < 0.001, ****p < 0.0001.

ELISA confirmed elevated CLU protein concentrations in both serum and CSF from MS patients compared to controls (Figure 4F). In vitro, combined cytokine and DNA stimulation significantly upregulated *Clu* mRNA expression and CLU protein secretion from primary astrocytes (Figure 4G,H). Recombinant CLU protein alone induced oligodendrocyte death comparable to conditioned medium from stimulated astrocytes (Figure 4I), establishing CLU as a direct neurotoxic factor. Western blotting and immunofluorescence confirmed increased CLU expression in EAE spinal cords, with prominent colocalization within GFAP‐positive astrocytes in demyelinated white matter (Figure 4J,K). Notably, confocal imaging revealed spatial colocalization of CLU with P‐STING within astrocytes in EAE lesions (Figure S3D), linking pathway activation to effector secretion.

### 3.5 STING Inhibition Attenuates Astrocyte‐Mediated Neurotoxicity

To determine whether cGAS–STING signaling is necessary for astrocyte‐induced neuroinflammation, we employed the specific STING inhibitor H‐151. Pre‐treatment with H‐151 abolished STING phosphorylation in cytokine and DNA‐stimulated astrocytes and significantly reduced C3 expression (Figure 5A,B and Figure S4A). RNA‐seq analysis of H‐151‐treated astrocytes revealed downregulation of neuroinflammatory gene programs, including chemokine signaling, viral response pathways, and apoptotic signaling (Figure 5C and Figure S4B). Quantitative analyses confirmed reduced Ccl2, Ccl5, and *Clu* mRNA levels, as well as decreased CLU protein secretion (Figure 5D–F).

**FIGURE 5:**
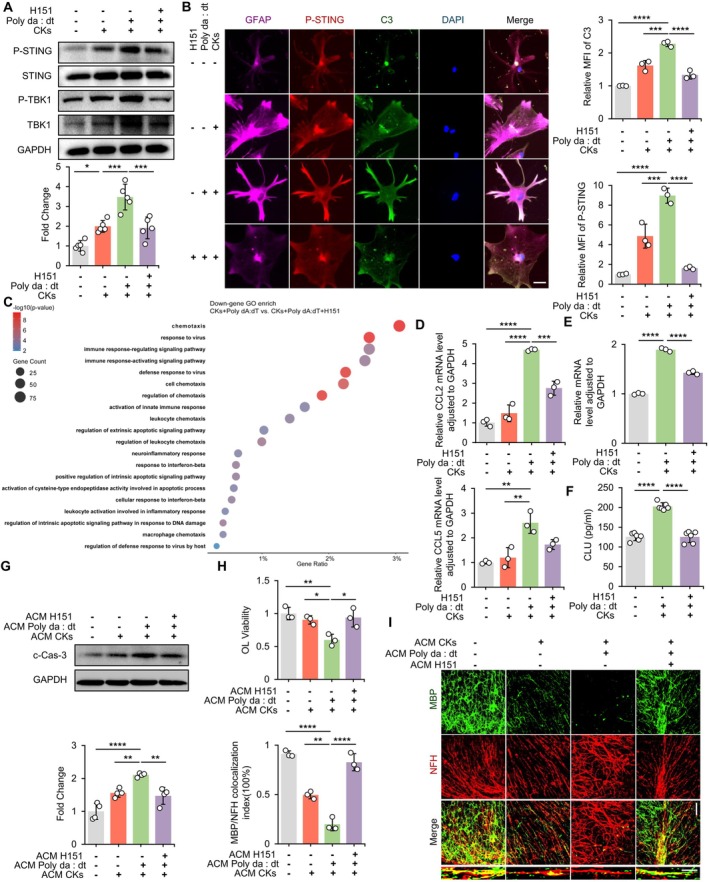
STING inhibitor H‐151 suppresses astrocyte‐mediated myelin damage. (A) Western blot analysis of cGAS‐STING pathway activation in primary mouse astrocytes pre‐treated with STING inhibitor H‐151 (10 μM) for 2 h, followed by stimulation with pro‐inflammatory cytokines (CKs) and/or poly(dA:DT) as indicated. Lower panel shows quantification of P‐STING fold change. (B) Representative confocal immunofluorescence images of astrocytes stained for GFAP (magenta), P‐STING (red), and complement component C3 (green) under indicated treatment conditions (scale bars, 20 μm). Right panels display quantification of relative MFI for C3 and P‐STING. (C) GO enrichment of genes downregulated by H‐151 pretreatment versus CKs + Poly (dA:DT) stimulation. (D) Quantitative RT‐PCR analysis of chemokine (Ccl2, Ccl5) mRNA expression in astrocytes under indicated treatment conditions, normalized to *Gapdh*. (E) Quantitative RT‐PCR analysis of *Clu* mRNA expression in astrocytes under indicated treatment conditions, normalized to *Gapdh*. (F) ELISA measurement of secreted CLU protein in ACM from the indicated treatment groups. (G) Western blot analysis of c‐Cas‐3 in primary oligodendrocytes treated for 24 h with ACM from the indicated astrocyte treatment groups, with densitometric quantification of fold change. (H) CCK‐8 assay of primary oligodendrocytes following 24 h treatment with ACM from the indicated groups. (I) Representative confocal immunofluorescence images of cerebellar organotypic slices treated with ACM from the indicated groups, stained for MBP (green) and NFH (red) (scale bar, 50 μm and 20 μm). Right panel shows quantification of MBP/NFH colocalization index. *p < 0.05, **p < 0.01, ***p < 0.001, ****p < 0.0001.

Functionally, conditioned medium from H‐151‐treated astrocytes showed markedly reduced neurotoxic potential, evidenced by decreased oligodendrocyte apoptosis (c‐Cas‐3 expression), improved cell viability, and preserved myelin integrity in cerebellar slice cultures (Figure 5G–I). These data demonstrate that STING inhibition disrupts the astrocyte‐mediated neurotoxic cascade.

### 3.6 STING Inhibition Ameliorates Clinical and Pathological Outcomes in EAE

To evaluate therapeutic potential in vivo, we administered H‐151 to EAE mice via daily intraperitoneal injection in either preventive (starting day 8 post‐immunization, prior to symptom onset) or therapeutic (starting at clinical onset) regimens. Both treatment paradigms significantly attenuated clinical disease severity compared to vehicle controls (Figure 6A). Luxol fast blue staining revealed reduced demyelinated lesion areas in H‐151‐treated spinal cords (Figure 6B).

**FIGURE 6:**
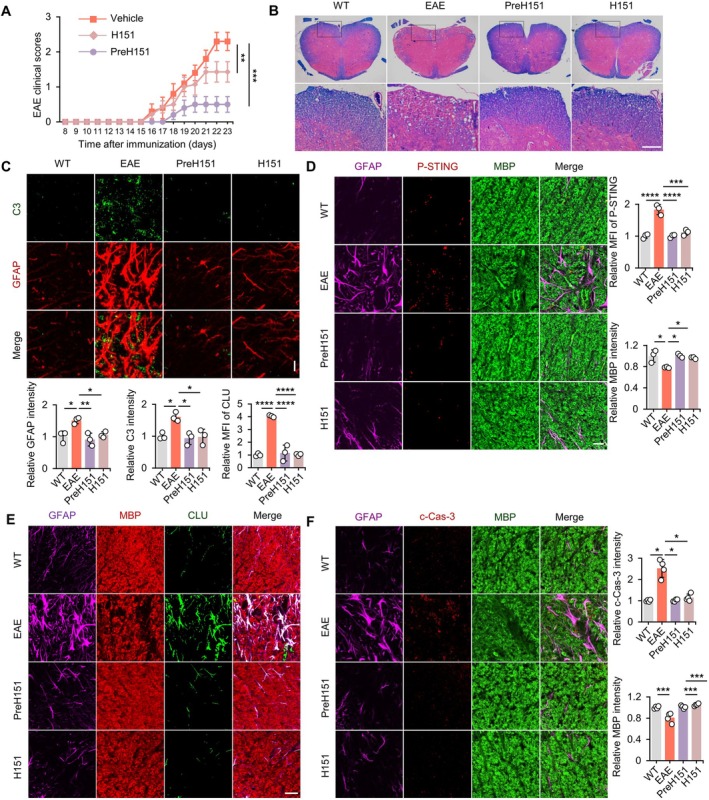
STING inhibitor H‐151 ameliorates clinical severity and central nervous system pathology in EAE mouse model. (A) Clinical disease course of EAE mice treated with vehicle, preventive H‐151 (PreH151; 750 nmol/mouse daily intraperitoneal injection from day 8 post‐immunization, prior to symptom onset), or therapeutic H‐151 (H151; from clinical symptom onset to disease plateau). Disease scores were recorded daily using a standardized 0–5 scale. (B) Representative Luxol fast blue–hematoxylin and eosin (LFB‐HE) staining of spinal cord cross‐sections from WT, EAE, PreH151, and H151 groups, showing demyelinated areas (pale staining) in the white matter (upper panels, scale bars 200 μm; lower magnified panels, scale bars 50 μm). (C) Representative confocal immunofluorescence images of lumbar spinal cord white matter stained for GFAP (magenta) and complement component C3 (green), with quantification of relative MFI for GFAP, C3, and CLU (scale bars, 20 μm). (D) Immunofluorescence images of P‐STING (red) in GFAP+ astrocytes (magenta), with MBP (green) counterstaining to identify demyelinated regions (scale bars, 20 μm). Right panels show quantification of P‐STING and MBP MFI. (E) Representative images showing CLU (green) expression in GFAP+ astrocytes (magenta) with MBP (red) counterstaining. up panels show quantification of CLU MFI (scale bars, 20 μm). (F) Immunofluorescence images of c‐Cas‐3 (red) in GFAP+ astrocyte–adjacent cells (magenta) with MBP (green) counterstaining. Right panels show quantification of c‐Cas‐3 and MBP MFI (scale bars, 20 μm). *p < 0.05, **p < 0.01, ***p < 0.001, ****p < 0.0001.

Western blotting of spinal cord lysates confirmed reduced P‐STING, C3, and GFAP expression in treated mice (Figure S5A). Immunofluorescence analysis demonstrated decreased C3 and P‐STING immunoreactivity within GFAP‐positive astrocytes (Figure 6C,D), accompanied by reduced CLU expression and P‐STING/CLU colocalization (Figure 6E and Figure S5B). H‐151 treatment also reduced spinal cord CLU protein concentrations (Figure S5C‐D) and diminished immune cell infiltration (CD45+ and F4/80+ cells) (Figure S5E). Notably, oligodendrocyte apoptosis (c‐Cas‐3) was reduced while MBP expression increased in treated mice (Figure 6F). Collectively, these findings establish that pharmacological STING inhibition mitigates neuroinflammation and demyelination by suppressing astrocytic CLU‐mediated neurotoxicity.

## 4 Discussion

This study establishes the DNA‐sensing cGAS‐STING pathway in astrocytes as a critical molecular link between innate immune surveillance and progressive demyelination in MS. Our findings demonstrate that astrocytes function as pivotal sensors of CNS‐derived DNA danger signals, integrating cytosolic DNA detection with pro‐inflammatory priming to drive a robust neurotoxic transformation. Through combined transcriptomic, single‐cell, and functional analyses, we identify CLU as a distinct, non‐canonical effector of this response that directly mediates oligodendrocyte apoptosis. Pharmacological targeting of STING disrupts this deleterious cascade, highlighting a therapeutic strategy to mitigate CNS‐intrinsic neurodegeneration independent of peripheral immunomodulation.

These findings extend the current understanding of astrocyte contributions to neuroinflammation beyond traditional cytokine‐mediated mechanisms. While the cGAS‐STING pathway has emerged as a key regulator of neuroinflammatory responses, mechanistic studies have historically emphasized microglial activation, wherein DNA sensing‐driven STING signaling promotes type I interferon production and inflammasome‐dependent cell death. For example, in ischemic stroke and sepsis‐associated encephalopathy models, STING activation in microglia exacerbates neuroinflammation through pyroptotic cell death [24, 25].

However, accumulating evidence indicates that astrocytes possess autonomous DNA‐sensing capabilities that amplify inflammatory output. In neuromyelitis optica spectrum disorder (NMOSD), mitochondrial DNA leakage activates the astrocytic cGAS‐STING pathway, driving A1‐like inflammatory polarization [26]. Our data expand this framework by demonstrating that exogenous DNA likely derived from damaged neurons or oligodendrocytes in the context of demyelinating injury synergizes with cytokine signaling to trigger a self‐perpetuating neurotoxic cascade. This “two‐hit” model provides a mechanistic basis for the clinical observation that disability progression in MS often continues despite suppression of peripheral immune cell infiltration, as compartmentalized CNS inflammation sustained by DNA release from cumulative tissue damage drives ongoing astrocyte‐mediated injury.

A key advance of this study is the identification of CLU as a major downstream mediator of astrocyte‐induced oligodendrocyte death. While canonical cGAS‐STING activation is traditionally associated with type I interferon and NF‐κB‐dependent cytokine programs [27], our transcriptomic and functional analyses reveal that, in the specific context of demyelinating lesions, CLU secretion represents a critical, previously unrecognized neurotoxic output. This positions CLU as a non‐canonical STING effector that operates in parallel to traditional interferon responses.

The neurotoxic potential of astrocyte‐derived CLU aligns with recent evidence implicating CLU in glial pathology [28, 29]. While CLU has been traditionally studied as a chaperone with neuroprotective properties under homeostatic conditions, our findings support a “toxic gain‐of‐function” hypothesis, wherein chronic inflammatory stress within the MS microenvironment alters CLU conformation or compartmentalization, converting it into a mediator of oligodendrocyte membrane compromise. Notably, single‐cell analysis revealed elevated CLU expression specifically in reactive astrocytes at chronic active lesion edges establishing a spatial correlation between CLU production and active demyelination.

While our integrated transcriptomic and functional analyses identify CLU as a distinct, non‐canonical secreted effector of astrocytic cGAS–STING signaling in the context of demyelination, we do not claim it to be the sole mediator. Canonical STING activation induces type I interferons (e.g., IFN‐β), NF‐κB‐dependent pro‐inflammatory cytokines (e.g., TNF‐α), and chemokines (e.g., CCL2, CCL5, CXCL10). Our ELISA and functional data indicate that IFN‐β, although the most abundantly secreted canonical STING product, exerts modest direct toxicity on oligodendrocytes compared with CLU. However, IFN‐β and CLU act synergistically to amplify oligodendrocyte injury. Thus, CLU functions as the dominant neurotoxic effector within the STING‐driven secretory program.

From a therapeutic perspective, we show that inhibition of the STING pathway with the small‐molecule inhibitor H‐151 significantly alleviates EAE severity. These data also underscore the need to distinguish between systemic and CNS‐intrinsic interferon signaling in MS. While systemic IFN‐β administration remains a therapy that modulates peripheral T‐cell subsets [30, 31], our study and recent literature indicate that chronic, localized IFN signaling within the CNS predominantly of glial origin—impairs remyelination and exacerbates neurotoxicity. Therefore, therapeutic strategies should differentiate systemic immunomodulation from approaches aimed at suppressing intrinsic CNS inflammation. In this context, our findings suggest that targeting STING may provide a focused approach to dampening the local neuroinflammatory milieu without compromising systemic immunity.

Finally, the cell‐type specificity of our findings should be considered within a broader neuroimmune context. Because cGAS–STING signaling is also highly active in microglia during neurodegeneration, and because we used the systemic inhibitor H‐151 rather than astrocyte‐specific STING conditional knockout mice, the therapeutic benefit observed in vivo likely reflects combined suppression of STING signaling across multiple cell types, including microglia, astrocytes, and potentially neurons. Accordingly, results obtained with systemic administration should be interpreted cautiously, acknowledging that the observed neuroprotection may arise from multicellular regulation. Nevertheless, together with our in vitro evidence that conditioned medium from STING‐activated astrocytes is sufficient to induce oligodendrocyte death, and the lesion‐associated astrocyte‐specific upregulation of CLU, our data support an important role for the astrocytic cGAS–STING axis in driving myelin injury.

## Author Contributions

Shengfei Hu: formal analysis (equal); investigation (equal); writing – original draft (equal). Xia Xiao: formal analysis (equal); investigation (equal). Xi Cheng: formal analysis (equal); investigation (equal). Yiying Huang: investigation (equal). Tingting Cui: investigation (equal). Shishi Shen: investigation (equal). Chunping Cui: investigation (equal). Hanzhang Tan: investigation (equal). Wei Qiu: supervision (equal). Yipeng Zhao: conceptualization (equal); funding acquisition (equal); methodology (equal) writing – review and editing (equal). Rui Li: conceptualization (equal); funding acquisition (equal); methodology (equal); writing – review and editing (equal).

## Funding

This work was supported by National Natural Science Foundation of China, U24A20691, 82271377, 81901229. Basic and Applied Basic Research Foundation of Guangdong Province, 2022B1515120042. Guangzhou Municipal Science and Technology Project, 2025B03J0035.

## Ethics Statement

The study protocol was approved by the Ethics Committee of the Third Affiliated Hospital of Sun Yat‐sen University (RG2024‐187‐01) and conducted in accordance with the Declaration of Helsinki.

## Consent

The authors have nothing to report.

## Conflicts of Interest

The authors declare no conflicts of interest.

## Supporting information


**Figure S1:** Validation of cGAS–STING pathway activation in inflammatory astrocytes in vitro and in vivo. (A) RNA‐seq data analysis of cGAS‐STING pathway component relative expression (*Cgas, Sting, Tbk1, Irf3*) in primary mouse astrocytes treated for 24 h with CKs versus PBS control (Ctrl). (B) Western blot analysis of cGAS protein expression in primary astrocytes following 24‐h CKs stimulation, with Vinculin as loading control. Right panel shows densitometric quantification of fold change. (C) Western blot analysis of cGAS, P‐STING and total STING protein levels in spinal cord lysates from WT and EAE mice, with GAPDH as loading control. Lower panels show densitometric quantification of CGAS and P‐STING fold change. (D) Representative confocal immunofluorescence images of lumbar spinal cord white matter from WT and EAE mice stained for GFAP (magenta), cGAS (red), and MBP (green). Right panel shows quantification of cGAS relative MFI. *p < 0.05.
**Figure S2:** Temporal dynamics of cGAS–STING activation and exacerbation of astrocyte‐mediated neurotoxicity. (A) Time‐course Western blot analysis of cGAS‐STING pathway activation in primary mouse astrocytes stimulated with CKs for 24 h followed by poly (dA:dT) treatment for indicated durations (0–24 h). Proteins detected include cGAS, P‐STING, and total STING, with Vinculin as loading control. Right panel shows quantification of P‐STING fold change over time. (B) Western blot analysis of complement component C3 expression in primary astrocytes under Ctrl, CKs alone, or combined CKs + poly(dA:dT) stimulation conditions, with GAPDH as loading control. Right panel shows densitometric quantification. (C) Representative immunofluorescence images of cerebellar organotypic slices treated for 24 h with ACM from the indicated stimulation groups, stained for MBP, (green) to assess myelin integrity (scale bar, 500 μm). Right panel shows quantification of relative MBP fluorescence intensity. *p < 0.05, **p < 0.01, ****p < 0.0001.
**Figure S3:** CLU is the principal STING‐driven neurotoxic effector among astrocyte‐secreted factors. Bulk RNA‐seq analysis of the top 20 upregulated genes in primary astrocytes stimulated with CKs + poly(dA:dT) versus Ctrl. ELISA quantification of canonical STING downstream factors (CCL2, CCL5, CXCL10, TNF‐α, and IFN‐β) in astrocyte‐conditioned medium from Ctrl and CKs + poly(dA:dT)‐stimulated astrocytes. CCK‐8 assay of primary oligodendrocyte viability after treatment with recombinant IFN‐β protein (200 ng/mL), recombinant CLU protein (100 ng/mL), or both. Representative confocal immunofluorescence images of lumbar spinal cord white matter from WT and EAE mice, showing triple staining for GFAP (magenta), P‐STING (red), and CLU (green) (scale bars, 20 μm). Right panel shows quantification of CLU relative MFI.
**Figure S4:** H‐151 reverses inflammatory marker expression and chemokine profiles in stimulated astrocytes. (A) Western blot analysis of complement component C3 expression in primary mouse astrocytes under indicated treatment conditions: Ctrl, CKs alone, CKs + poly (dA:dT), and CKs + poly(dA:dT) with H‐151 pre‐treatment. GAPDH served as loading control. Right panel shows densitometric quantification of C3 fold change. (B) Heatmap visualization of chemokine gene expression profiles in astrocytes stimulated with CKs + poly(dA:dT) versus those pre‐treated with H‐151 prior to stimulation. Color scale indicates Z‐score normalized expression levels. *p < 0.05, **p < 0.01, ***p < 0.001, ****p < 0.0001.
**Figure S5:** In vivo effects of H‐151 on STING signaling, immune cell infiltration, and CLU production in EAE. (A) Western blot analysis of cGAS‐STING pathway components (cGAS, P‐STING, total STING), complement component C3, and GFAP in spinal cord lysates from WT, EAE, PreH151, and H‐151 groups, with GAPDH as loading control. Right panels show densitometric quantification of cGAS, P‐STING, C3, and GFAP fold change. (B) Representative confocal immunofluorescence images of lumbar spinal cord white matter stained for GFAP (magenta), P‐STING (red), and CLU (green), with merged images (scale bars, 20 μm). Right panel shows quantification of CLU relative MFI. (C) Western blot analysis of CLU protein expression in spinal cord lysates from the indicated groups, with GAPDH as loading control. Lower panel shows densitometric quantification. (D) ELISA measurement of CLU protein concentration in spinal cord homogenates from WT, EAE, PreH151, and H151 groups. (E) Representative immunofluorescence images of spinal cord cross‐sections stained for CD45 (magenta, lymphocyte marker), and F4/80 (red, macrophage/microglia marker), showing immune cell infiltration patterns across treatment groups (upper panels, scale bars 200 μm; lower magnified panels, scale bars 50 μm). *p < 0.05, **p < 0.01, ***p < 0.001, ****p < 0.0001.

## Data Availability

The data that support the findings of this study are available from the corresponding author upon reasonable request.
