# Supplementary material for: The cGAS–STING Pathway Drives Astrocyte‐Mediated Demyelination in Multiple Sclerosis Through Clusterin Secretion
Source: CNS Neurosci Ther. 2026 Aug 14;32(8):e71091. doi: 10.1002/cns.71091 (PMC13476701; doi:10.1002/cns.71091)
Supplement: Supplementary file 1 [file CNS-32-e71091-s001.docx]

**Figure S1. Validation of cGAS–STING pathway activation in inflammatory astrocytes in vitro and in vivo**

**(A)** RNA-seq data analysis of cGAS-STING pathway component relative expression (*Cgas, Sting, Tbk1, Irf3*) in primary mouse astrocytes treated for 24 h with CKs versus PBS control (Ctrl).

**(B**) Western blot analysis of cGAS protein expression in primary astrocytes following 24-hour CKs stimulation, with Vinculin as loading control. Right panel shows densitometric quantification of fold change.

**(C)** Western blot analysis of cGAS, P-STING and total STING protein levels in spinal cord lysates from WT and EAE mice, with GAPDH as loading control. Lower panels show densitometric quantification of CGAS and P-STING fold change.

**(D)** Representative confocal immunofluorescence images of lumbar spinal cord white matter from WT and EAE mice stained for GFAP (magenta), cGAS (red), and MBP (green). Right panel shows quantification of cGAS relative MFI.

**P* < 0.05.

**Figure S2. Temporal dynamics of cGAS–STING activation and exacerbation of astrocyte-mediated neurotoxicity**

(A) Time-course Western blot analysis of cGAS-STING pathway activation in primary mouse astrocytes stimulated with CKs for 24 h followed by poly (dA:dT) treatment for indicated durations (0–24 h). Proteins detected include cGAS, P-STING, and total STING, with Vinculin as loading control. Right panel shows quantification of P-STING fold change over time.

(B) Western blot analysis of complement component C3 expression in primary astrocytes under Ctrl, CKs alone, or combined CKs + poly(dA:dT) stimulation conditions, with GAPDH as loading control. Right panel shows densitometric quantification.

(C) Representative immunofluorescence images of cerebellar organotypic slices treated for 24 h with ACM from the indicated stimulation groups, stained for MBP, (green) to assess myelin integrity (scale bar, 500 μm). Right panel shows quantification of relative MBP fluorescence intensity.

**P* < 0.05, ***P* < 0.01, *****P* < 0.0001.

**Figure S3. CLU is the principal STING-driven neurotoxic effector among astrocyte-secreted factors.**

(A) Bulk RNA-seq analysis of the top 20 upregulated genes in primary astrocytes stimulated with CKs + poly(dA:dT) versus Ctrl.
(B) ELISA quantification of canonical STING downstream factors (CCL2, CCL5, CXCL10, TNF-α, and IFN-β) in astrocyte-conditioned medium from Ctrl and CKs + poly(dA:dT)-stimulated astrocytes.
(C) CCK-8 assay of primary oligodendrocyte viability after treatment with recombinant IFN-β protein (200ng/mL), recombinant CLU protein (100ng/mL), or both.
(D) Representative confocal immunofluorescence images of lumbar spinal cord white matter from WT and EAE mice, showing triple staining for GFAP (magenta), P-STING (red), and CLU (green) (scale bars, 20 μm). Right panel shows quantification of CLU relative MFI.

**Figure S4. H-151 reverses inflammatory marker expression and chemokine profiles in stimulated astrocytes**

**(A)** Western blot analysis of complement component C3 expression in primary mouse astrocytes under indicated treatment conditions: Ctrl, CKs alone, CKs + poly (dA:dT), and CKs + poly(dA:dT) with H-151 pre-treatment. GAPDH served as loading control. Right panel shows densitometric quantification of C3 fold change.

**(B)** Heatmap visualization of chemokine gene expression profiles in astrocytes stimulated with CKs + poly(dA:dT) versus those pre-treated with H-151 prior to stimulation. Color scale indicates Z-score normalized expression levels.

**P* < 0.05, ***P* < 0.01, ****P* < 0.001, *****P* < 0.0001.

**Figure S5.** **In vivo effects of H-151 on STING signaling, immune cell infiltration, and CLU production in EAE**

(A) Western blot analysis of cGAS-STING pathway components (cGAS, P-STING, total STING), complement component C3, and GFAP in spinal cord lysates from WT, EAE, PreH151, and H-151 groups, with GAPDH as loading control. Right panels show densitometric quantification of cGAS, P-STING, C3, and GFAP fold change.

**(B)** Representative confocal immunofluorescence images of lumbar spinal cord white matter stained for GFAP (magenta), P-STING (red), and CLU (green), with merged images (scale bars, 20 μm). Right panel shows quantification of CLU relative MFI.

**(C)** Western blot analysis of CLU protein expression in spinal cord lysates from the indicated groups, with GAPDH as loading control. Lower panel shows densitometric quantification.

**(D)** ELISA measurement of CLU protein concentration in spinal cord homogenates from WT, EAE, PreH151, and H151 groups.

**(E)** Representative immunofluorescence images of spinal cord cross-sections stained for CD45 (magenta, lymphocyte marker), and F4/80 (red, macrophage/microglia marker), showing immune cell infiltration patterns across treatment groups (upper panels, scale bars 200 μm; lower magnified panels, scale bars 50 μm).

**P* < 0.05, ***P* < 0.01, ****P* < 0.001, *****P* < 0.0001.
